# SMYD3 Promotes Homologous Recombination via Regulation of H3K4-mediated Gene Expression

**DOI:** 10.1038/s41598-017-03385-6

**Published:** 2017-06-19

**Authors:** Yun-Ju Chen, Cheng-Hui Tsai, Pin-Yu Wang, Shu-Chun Teng

**Affiliations:** 1 Department of Microbiology, College of Medicine, National Taiwan University, Taipei, 10051 Taiwan; 2 Ph.D. Program in Translational Medicine, National Taiwan University and Academia Sinica, Taipei, 10051 Taiwan

## Abstract

SMYD3 is a methyltransferase highly expressed in many types of cancer. It usually functions as an oncogenic protein to promote cell cycle, cell proliferation, and metastasis. Here, we show that SMYD3 modulates another hallmark of cancer, DNA repair, by stimulating transcription of genes involved in multiple steps of homologous recombination. Deficiency of SMYD3 induces DNA-damage hypersensitivity, decreases levels of repair foci, and leads to impairment of homologous recombination. Moreover, the regulation of homologous recombination-related genes is via the methylation of H3K4 at the target gene promoters. These data imply that, besides its reported oncogenic abilities, SMYD3 may maintain genome integrity by ensuring expression levels of HR proteins to cope with the high demand of restart of stalled replication forks in cancers.

## Introduction

Cells inevitably encounter the challenge of chromosomal double-strand breaks (DSBs) during their lifetime. The oxidative byproducts of the normal metabolic process and exogenous factors such as chemical agents or ionizing radiation (IR) constantly threaten the integrity of our genome. Unrepaired or misrepaired DNA lesions can lead to genome instability, which is a hallmark of cancer^1^. Two major pathways, homologous recombination (HR) and non-homologous end joining (NHEJ), are responsible for repairing these breaks.

HR occurs predominantly at S and G2 phases when a sister chromatid is accessible^2^. The repair is initiated by a resection process, which includes MRE11-RAD50-NBS1 (MRN) end sensing complex, CtIP endonuclease^3^, EXO1 exonuclease^4^, and BLM helicase^5^, to remove oligonucleotides from each side of the DSB and expose single-stranded DNA (ssDNA) tails for forming RAD51-ssDNA filaments with the help of BRCA2^6^. Working in concert with RAD51-ssDNA filaments, RAD54B, a DNA-dependent ATPase, drives the search for a homologous template and strand invasion^7, 8^, which leads to accurate repair. Classical NHEJ (C-NHEJ) occurs throughout the cell cycle but predominately at G1 phase. During C-NHEJ, cells utilise Ku70/Ku80 heterodimer and DNA-dependent protein kinase (DNA-PK) to recognize and ligate DSB ends via little (less than ten base pairs) or no homology between the joined ends, which is, therefore, an error-prone pathway^9, 10^. Besides, alternative end-joining pathways, such as microhomology-mediated end joining (MMEJ), do not use Ku- and DNA-PK. Initial resection produces relatively longer stretches of microhomology (5–25 base pairs), and subsequent flap trimming and end-joining often create the final mutagenic MMEJ repair products^11^.

DSB repair is facilitated through chromatin modifications to open the compact barriers and improve the accessibility of repair proteins^12^. For example, the rapid phosphorylation of H2A.X at S139 in mammals (forming γH2A.X) by ATM within minutes at DSB sites is considered as a major hallmark of DSB recognition^13, 14^. γH2A.X further interacts with the mammalian repair mediator MDC1. MDC1 recruits RNF8^15^ and RNF168^16^ to catalyze K63-ubiquitilation on H2A and H2A.X to recruit BRCA1 and 53BP1 for HR and NHEJ, respectively^15, 17–19^. While the posttranslational modifications of proteins in DSB repair have been broadly studied^20, 21^, the evidence of transcriptional regulation of DSB repair proteins is comparatively scarce.

Cancer development is closely related to aberrant histone modification which causes abnormal genes expression. Many histone methyltransferases have been implicated in cancer aggressiveness^22, 23^. SMYD3 methyltransferase is highly expressed in colorectal carcinomas, hepatocellular carcinomas, pancreatic cancer, prostate cancer, and breast cancer^24, 25^. SMYD3 regulates gene transcription through methylating histones, including H2.A.ZK101me2^26^, H3K4me2/3^27^, H4K20me2/3^28^, and H4K5me1/2/3^29^. For example, SMYD3 methylates H2A.Z to activate cyclin A1 expression and drive cancer proliferation^26^, and H3K4 to upregulate MMP9^30^ and hTERT expression^31^. Moreover, SMYD3 modifies non-histone proteins VEGFR and MAP3K2 to promote metastasis^32^ and Ras/Raf/MEK/ERK signaling^33^ in cancer development, respectively.

Previous studies have focused on the ability of how SMYD3 stimulates cell proliferation and metastasis. Here, we identify a new role of SMYD3 in regulating HR repair. Inhibition of SMYD3 directly blunts HR efficiency by downregulating the expression of HR-related genes. Additionally, SMYD3 knockdown leads to decreased methylation of H3K4 and recruitment of RNA polymerase II (RNAPII) at the target gene promoters. These data reveal that SMYD3 maintains genome stability by ensuring normal expression levels of HR repair proteins.

## Results

### Microarray data analysis identifies SMYD3-regulated expression of DNA repair machinery

**Figure 1:**
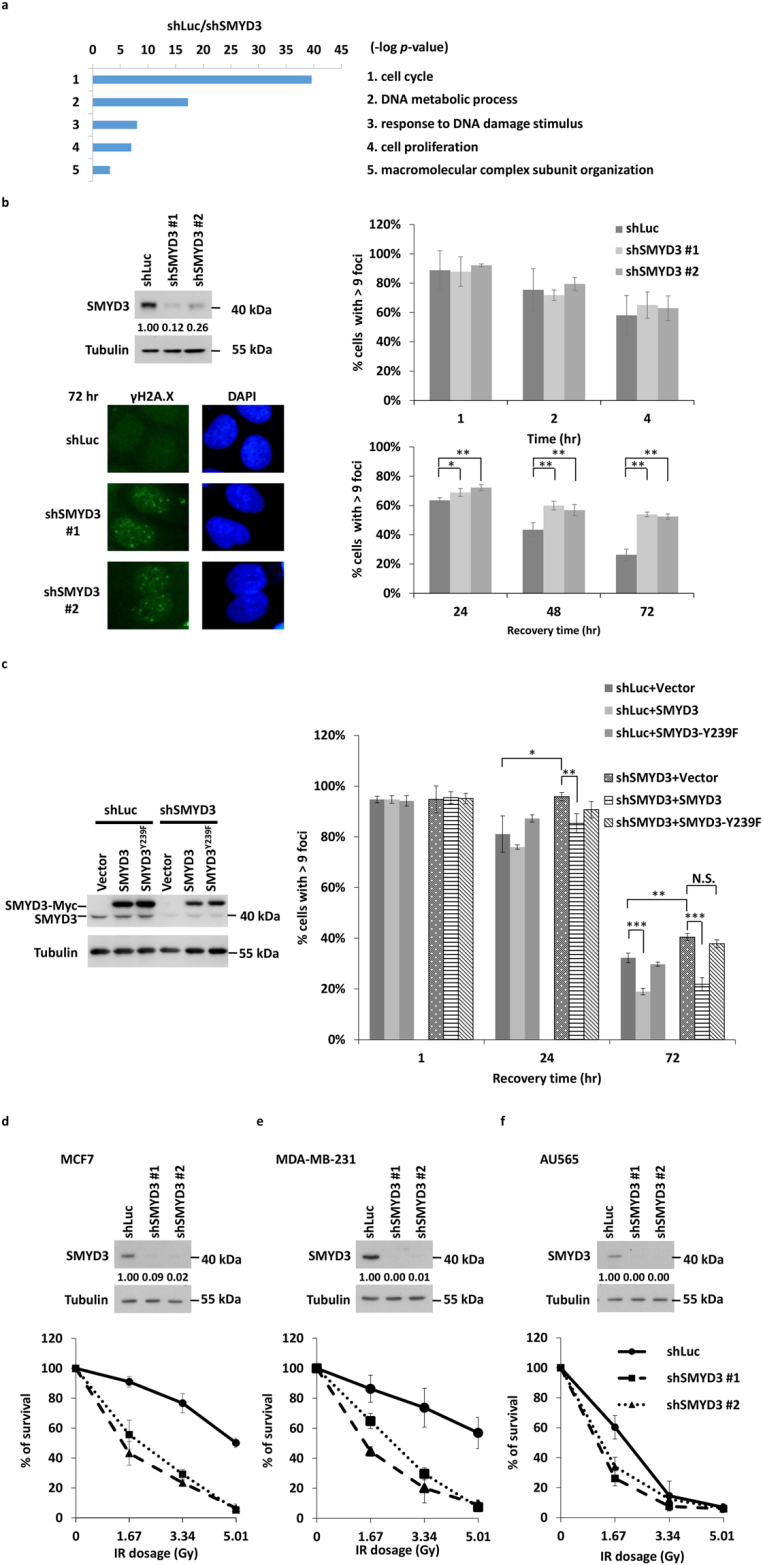
SMYD3 is required for DNA repair machinery. (**a**) Whole-genome microarray analysis of RNAs isolated from shLuc vs. shSMYD3 MCF7 cells was conducted. With a cut-off of absolute normalized fold change  $\leqq$ 0.5 (log2 normalized ratios < −1), the list of down-regulated genes was further categorized by the DAVID v6.8 Gene Ontology program to reveal their gene ontology process. (**b**) γH2A.X foci formation at indicated times after 1.67 Gy IR treatment in shLuc or shSMYD3 MCF7 cells. *P < 0.05. **P < 0.01. (**c**) γH2A.X foci formation at indicated times after 1.67 Gy IR treatment in shLuc or shSMYD3 MCF7 cells complemented with the vector control, SMYD3 or mutant SMYD3^Y239F^. *P < 0.05. **P < 0.01. ***P < 0.001. (**d**–**f**) Clonogenic survivals of shLuc and shSMYD3 MCF7 (**d**), MDA-MB-231 (**e**) and AU565 cells (**f**) treated with indicated dosages of IR. All values in the diagrams were means ± SD of triplicates and data were representative of n ≥ 3 for each experiment.

Increased expression of SMYD3 can promote cancer proliferation^24^ and metastasis^30^. To explore additional and novel roles of SMYD3 in biological processes, we analysed our previously conducted whole-genome microarray data of RNAs isolated from shLuc vs. shSMYD3 MCF7 cells (GEO accession number GSE58048), in which a lentivirus shRNA infection system was used for stable knockdown of SMYD3^26^. 449 genes were downregulated upon SMYD3 knockdown. The gene ontology (GO) analysis indicated that these genes were mainly involved in cell cycle, DNA metabolic process, response to DNA damage stimulus, cell proliferation and macromolecular complex subunit organization (Fig. 1a). Previous reports provided evidence that SMYD3-dependent histone methylations are essential for cell cycle and cell proliferation. Intriguingly, SMYD3 is associated with DNA damage response (DDR) in the top three categories of GO analysis. Since SMYD3 has not been linked to DDR or DNA repair, the mechanism was further analysed.

We first investigated whether SMYD3 knockdown cells are more vulnerable to DNA damage stress such as IR. To examine the repair rate following IR, the formation of γH2A.X foci were used as a marker for DNA damage. Following exposure to 1.67 Gy of IR, SMYD3 knockdown cells significantly delayed the removal of γH2A.X foci at 48 and 72 hr compared to the shLuc controls (Fig. 1b). Conversely, exogenous expression of SMYD3, but not the catalytic dead mutant SMYD3^Y239F^ proteins, in the SMYD3 knockdown cells significantly restored the defects at 24 and 72 hr compared to that in the shSMYD3 with the expression of the vector control (Fig. 1c). The clonogenic survival following exposure to IR was further examined, and knockdown of SMYD3 led to impeded formation of colonies (Fig. 1d). Similar to that in MCF7 cells, shSMYD3 MDA-MB-231 and AU565 cells were more vulnerable to IR stress (Fig. 1e,f).

We next analysed the effect of IR on SMYD3’s cellular location. The majority of SMYD3 is located in the cytoplasm^33^, and we wondered whether it would translocate into the nucleus upon DNA damage insults. Cells were exposed to increasing dosages of IR and assayed for the translocation of SMYD3 protein to the nucleus at 1 and 3 hours. The distribution of SMYD3 did not show any noticeable difference after IR treatment (Supplementary Fig. S1a). Furthermore, the gene and protein expression of SMYD3 were not augmented after IR treatment (Supplementary Fig. S1b). These results suggest that DNA damage does not modulate SMYD3 expression and location.

**Figure 2:**
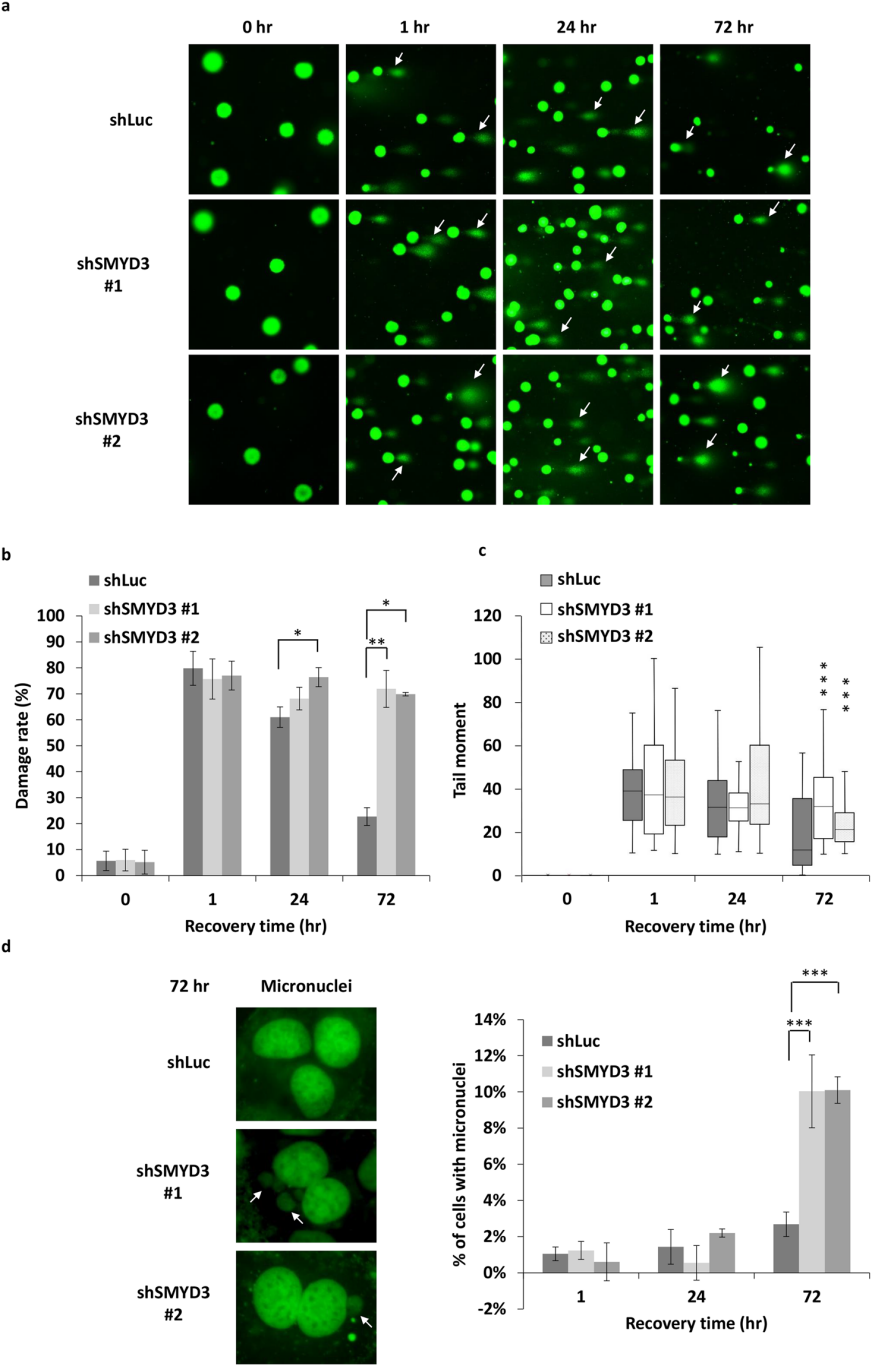
SMYD3 deficiency causes genome instability. (**a**,**b**) shLuc and shSMYD3 MCF7 cells were treated with 1.67 Gy IR and then analysed for DNA damage by comet assay at indicated times. (**a**) Representative images with white arrows point to comet tails, which signify DNA damage. (**b**) Quantification of the percentage of nuclei with comet tails. (**c**) Quantification of comet tail moment using the CometScore software. Data are presented as a quantile box plot. The middle lines in boxes indicate the median; upper and lower box edges indicate the 25th and 75th percentiles; and bars indicate the 10th and 90th percentiles. Statistical analysis was performed by one-way analysis variance (ANOVA). ***P < 0.001. (**d**) Right panel, representative images of micronuclei in shLuc and shSMYD3 MCF7 cells treated with 1.67 Gy IR and recovered for 72 h. White arrows point to micronuclei, which signify aberrant chromosomal segregation. Left panel, quantification of micronuclei at indicated times. ***P < 0.001. A minimum of 100 cells per treatment group were analysed for each quantification. All values in the diagrams were means ± SD of triplicates and data were representative of n ≥ 3 for each experiment.

We further examined whether SMYD3 affects genome integrity in cells. To determine if the loss of SMYD3 is associated with increased DNA damage, we performed a single-cell gel electrophoresis (comet) assay. The comet assay revealed increases of damage rate and tail moment in shSMYD3 compared to those in shLuc cells at 72 h (Fig. 2a–c). We also investigated micronuclei formation, a well-established indicator for genome instability^34, 35^, which occurs through the aberrant segregation of chromosomes or acentric chromosomal fragments. Compared to the shLuc control, knockdown of SMYD3 caused an increase in IR-induced micronuclei at 72 h (Fig. 2d). Taken together, these data suggest a role of SMYD3 in DNA repair mechanism.

### SMYD3 mediates the HR pathway

**Figure 3:**
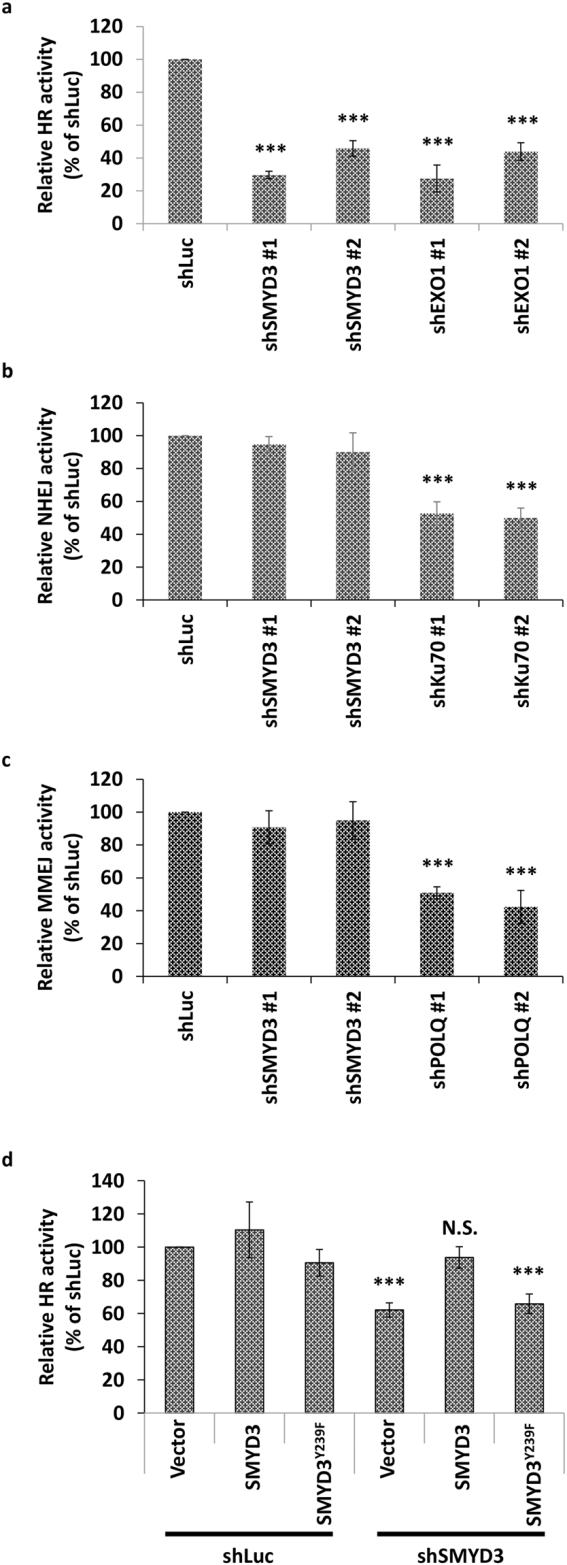
SMYD3 mediates the HR pathway. (**a**) Relative HR activity of shLuc, shSMYD3, and shEXO1 MCF7 cells, which was indicated by the percentage of GFP-positive cells relative to the shLuc control. The EXO1 knockdown cells were used as a positive control. ***P < 0.001 vs. shLuc control. (**b**) Relative NHEJ activity of shLuc, shSMYD3 and shKu70 cells. NHEJ activity was measured by normalizing the luciferase activity to the renilla activity. The Ku70-knockdown cells were used as a positive control. ***P < 0.001 vs. shLuc control. (**c**) Relative MMEJ activity of shLuc, shSMYD3 and shPOLQ MCF7 cells, which was indicated by the percentage of GFP-positive cells relative to the shLuc control. The POLQ-knockdown cells were used as a positive control. ***P < 0.001 vs. shLuc control. (**d**) Exogenous expression of SMYD3, but not the mutant SMYD3^Y239F^, restored the HR activity. ***P < 0.01 vs. shLuc with the expression of the vector plasmid control. N.S.: not significant.

Since mammalian DSB repair was achieved mainly by two mechanisms, HR and NHEJ, we wondered whether SMYD3 is involved in these pathways. We used cells with well-characterized GFP-based chromosomal reporters to detect the efficiency of HR. The reporter contains an I-SceI recognition sequence, which would be cleaved upon I-SceI expression to generate a DSB. DSB repair by HR using the direct repeat within the reporter cassette as a template results in an intact *GFP* gene. The repair efficiency was then quantified by flow cytometry. For the plasmid-based end-joining assay, a linearized plasmid harboring a luciferase reporter gene was used. Repair efficiency was measured by the luciferase activities of linearized reporter constructs compared with that of the intact plasmid. Results demonstrated that SMYD3 knockdown significantly hampered HR repair by 55–70% compared to the control cells (Fig. 3a). In contrast, SMYD3 knockdown did not change the NHEJ activity compared to the control cells (Fig. 3b). As the controls for the HR and NHEJ assays, knockdown of EXO1 reduced the HR activity and knockdown of Ku70 impaired the NHEJ activity by 56–73% and 47–50%, respectively (Fig. 3a,b). We also examined whether SMYD3 was required by MMEJ using a plasmid-based MMEJ assay and found that SMYD3 did not display any effect on the efficiency of MMEJ, while knockdown of the control, POLQ, reduced MMEJ activity by 49–58% (Fig. 3c). The knockdown effectiveness of each cell lines used was confirmed by qRT-PCR (Supplementary Fig. S2a–c). Moreover, the exogenous expression of SMYD3, but not the SMYD3^Y239F^ proteins (Supplementary Fig. S2d), restored the HR activity of the shSMYD3 cells (Fig. 3d). These results identify a role of SMYD3 in HR repair.

### SMYD3 knockdown downregulates HR gene expressions

**Figure 4:**
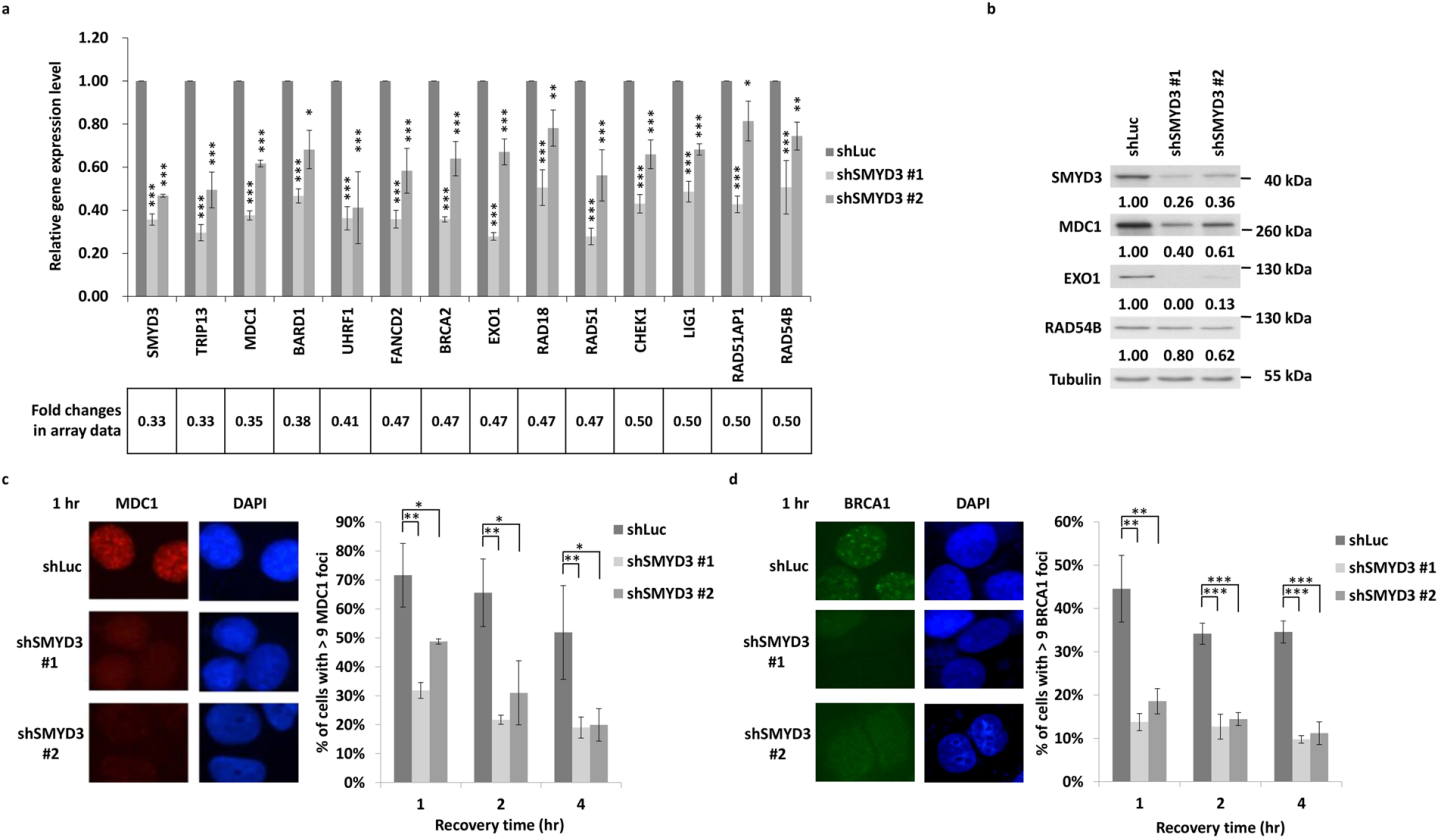
SMYD3 knockdown downregulates HR gene expressions. (**a**) The confirmation of the microarray analyses for the expressions of candidate genes in the shSMYD3/shLuc dataset using qRT-PCR. The fold changes of each gene expression in the microarray data were listed below. *P < 0.05, **P < 0.01, ***P < 0.001 vs. shLuc control. (**b**) Western blot analyses of SMYD3, MDC1, EXO1 and RAD54B in shLuc and shSMYD3 MCF7 cells. Tubulin was used as a loading control. The ratio of individual protein relative to the loading control tubulin was indicated below. (**c**,**d**) DNA repair foci formation of MDC1 foci (**c**) and BRCA1 foci (**d**) formation at indicated times after 1.67 Gy IR treatment in shLuc or shSMYD3 MCF7 cells. *P < 0.05. **P < 0.01. ***P < 0.001. All values in the histograms were means ± SD of triplicates and data were representative of n ≥ 3 for each experiment.

To understand the exact role of SMYD3 in HR repair, we analysed microarray-identified genes that were related to DDR and found that 25 of them are involved in DNA repair. Among these 25 genes, 13 genes are implicated in the HR pathway (Table 1). These genes range from the early step of DNA damage mediators, kinase transducer to downstream effectors that execute error-free repair process^36^. We performed qRT-PCR analysis to confirm their expressions and found that all genes exhibited similar fold differences in mRNA expression as initially identified in the microarray analysis (Fig. 4a).

**Table 1:** Down-regulated HR genes in shSMYD3/shLuc array data (genes with <0.5 fold differences).

ID	Log ratio	Ratio	Gene Title/Gene Symbol
218788_s_at	−1.6	0.33	SET and MYND domain containing 3/*SMYD3*
204033_at	−1.6	0.33	Thyroid hormone receptor interactor 13/*TRIP13*
203061_s_at	−1.5	0.35	Mediator of DNA-damage checkpoint 1/*MDC1*
205345_at	−1.4	0.38	BRCA1 associated RING domain 1/*BARD1*
225655_at	−1.3	0.41	Ubiquitin-like with PHD and ring finger domains 1/*UHRF1*
227545_at	−1.2	0.44	BRCA1 associated RING domain 1/*BARD1*
223545_at	−1.1	0.47	Fanconi anemia, complementation group D2/*FANCD2*
214727_at	−1.1	0.47	Breast cancer 2, early onset/*BRCA2*
204603_at	−1.1	0.47	Exonuclease 1/*EXO1*
238748_at	−1.1	0.47	RAD18 homolog (*S*. *cerevisiae*) /*RAD18*
205024_s_at	−1.1	0.47	RAD51 homolog (RecA homolog, *E*. *coli*) (*S*. *cerevisiae*)/*RAD51*
205394_at	−1.0	0.50	CHK1 checkpoint homolog (*S*. *pombe*)/*CHEK1*
242560_at	−1.0	0.50	Fanconi anemia, complementation group D2/*FANCD2*
202726_at	−1.0	0.50	Ligase I, DNA, ATP-dependent/*LIG1*
204146_at	−1.0	0.50	RAD51 associated protein 1/*RAD51AP1*
219494_at	−1.0	0.50	RAD54 homolog B (*S*. *cerevisiae*)/*RAD54B*

Among these genes, MDC1 plays the earliest role in HR repair^37^ and participates in the initial recruitment of BRCA1^38–42^ to promote DNA end resection for HR^43^. Moreover, EXO1 is the major exonuclease for efficient end resection^4^, and RAD54B is a DNA-dependent ATPase required for efficient chromatin remodeling during strand invasion^7, 8^. We checked the influence of SMYD3 depletion on MDC1, EXO1, and RAD54B. Consistently, the protein levels of MDC1 and EXO1 were significantly reduced after inhibition of SMYD3. Besides, a marginal reduction of the RAD54B protein was observed (Fig. 4b).

To gain further insight into how SMYD3 regulates HR activity, we investigated the significance of SMYD3 on the formation of MDC1 foci. After IR treatment, MDC1 foci were diminished in SMYD3-depleted cells at 1 to 4 hrs (Fig. 4c). And these MDC1 foci were disappeared at 24 to 72 hrs, even in the shLuc cells (Supplementary Fig. S3a). Moreover, after IR treatment, the formation of BRCA1 foci in SMYD3 knockdown cells was impaired as well (Fig. 4d and Supplementary Fig. S3b). In contrast, lack of SMYD3 did not affect the assembly of 53BP1 foci (Supplementary Fig. S3c). These data indicated that SMYD3 deficiency weakens HR partly through downregulation of MDC1, thereby compromising the recruitment of BRCA1 at DSBs.

### SMYD3 controls the expression of HR genes through methylating histone H3K4

**Figure 5:**
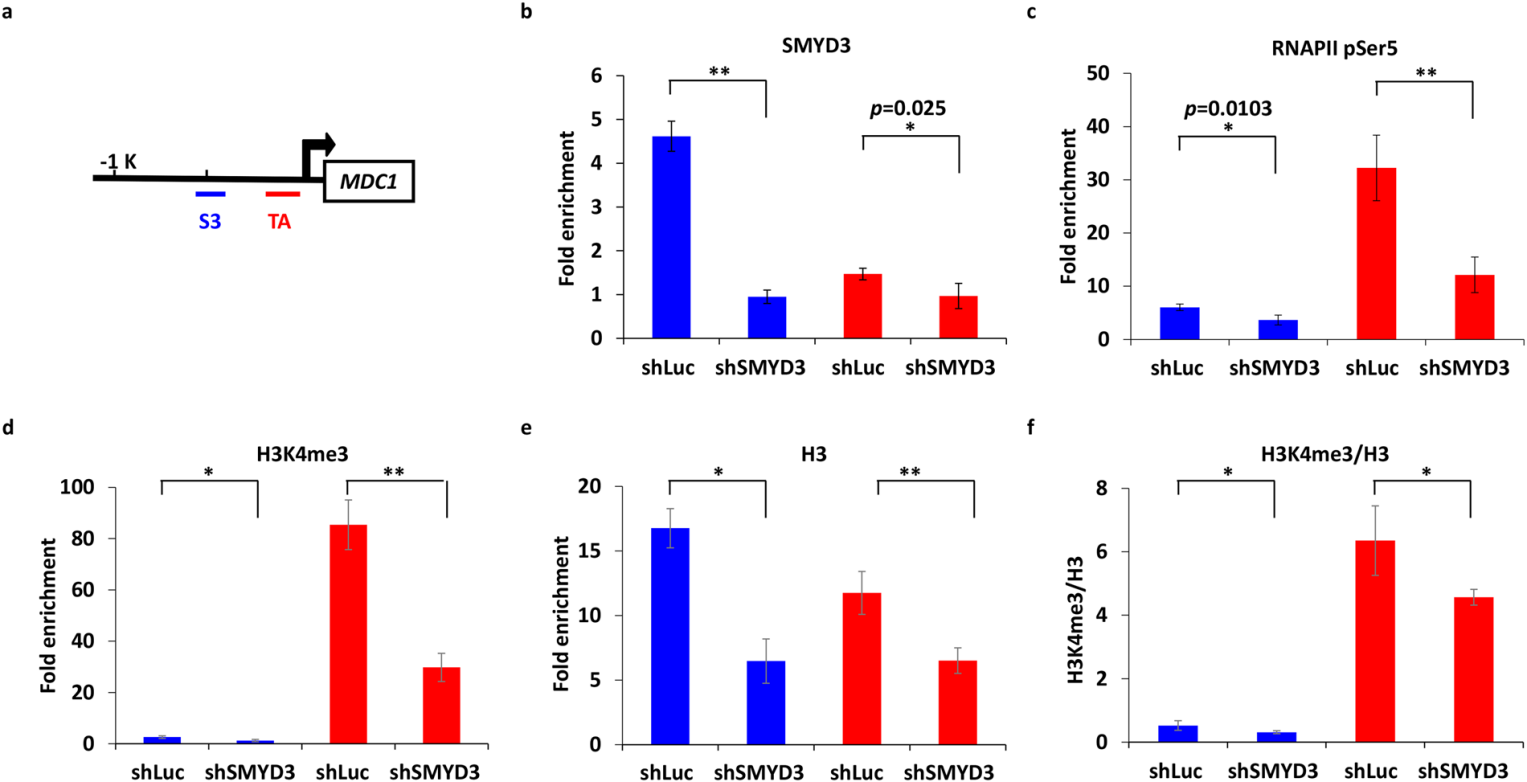
SMYD3 regulates the expression of *MDC1* through methylating histone H3K4. (**a**) ChIP assay was performed in MCF7 cells using specific antibodies. The examined positions at the *MDC1* locus were indicated, in which region S3 and region TA for the predicted SMYD3 and TATA box binding sites, respectively. (**b**–**e**) ChIP assays were performed with SMYD3-repressed MCF7 cells using specific antibodies shown at the top of the histogram. Fold enrichment of each antibody compared with IgG was shown. (**f**) Ratios of H3K4me3/H3 ChIP signals were displayed. In (**b**–**f**), immunoprecipitated chromatin was quantified by qRT-PCR. *P < 0.05. **P < 0.01. All values in the histograms were means ± SD of triplicates and data were representative of n ≥ 3 for each experiment.

SMYD3 was initially reported to methylate histone H3K4 to modulate the accessibility of chromatin architecture and to form a complex with RNA polymerase II (RNAPII) and RNA helicase HELZ to drive its target genes^24^. To explore the epigenetic regulation of SMYD3 on these HR genes, we examined the recruitments of SMYD3, H3K4me3, and RNAPII pSer5, which is a required RNAPII phosphorylation for the transcriptional initiation^44^, to the *MDC1*, *EXO1*, and *RAD54B* promoter regions. The putative TATA box (region TA) was retrieved from GPMiner^45^. ChIP experiments showed a direct binding of SMYD3 to the *MDC1* promoter (region S3) at ~500 bp upstream of the transcription start site (TSS) (Fig. 5a). SMYD3 preferred to enrich at region S3 (blue bar) than region TA (red bar) (Fig. 5b). In contrast, RNAPII pSer5 (Fig. 5c) and histone H3K4me3 (Fig. 5d) exhibited greater binding at region TA than region S3. Furthermore, knockdown of SMYD3 led to significant decreases of SMYD3, H3K4me3, H3 and RNAPII pSer5 at various degrees at both regions S3 and TA (Fig. 5b–e). Also, the enrichment of H3K4me3-modified histones (H3K4me3/H3) declined significantly in SMYD3 knockdown cells (Fig. 5f). A similar tendency was observed in the promoter regions of *EXO1* and *RAD54B* (Supplementary Fig. S4). These results suggest that SMYD3 may trigger HR gene expression by directly binding to the promoters to create an active histone mark for transcription.

## Discussion

**Figure 6:**
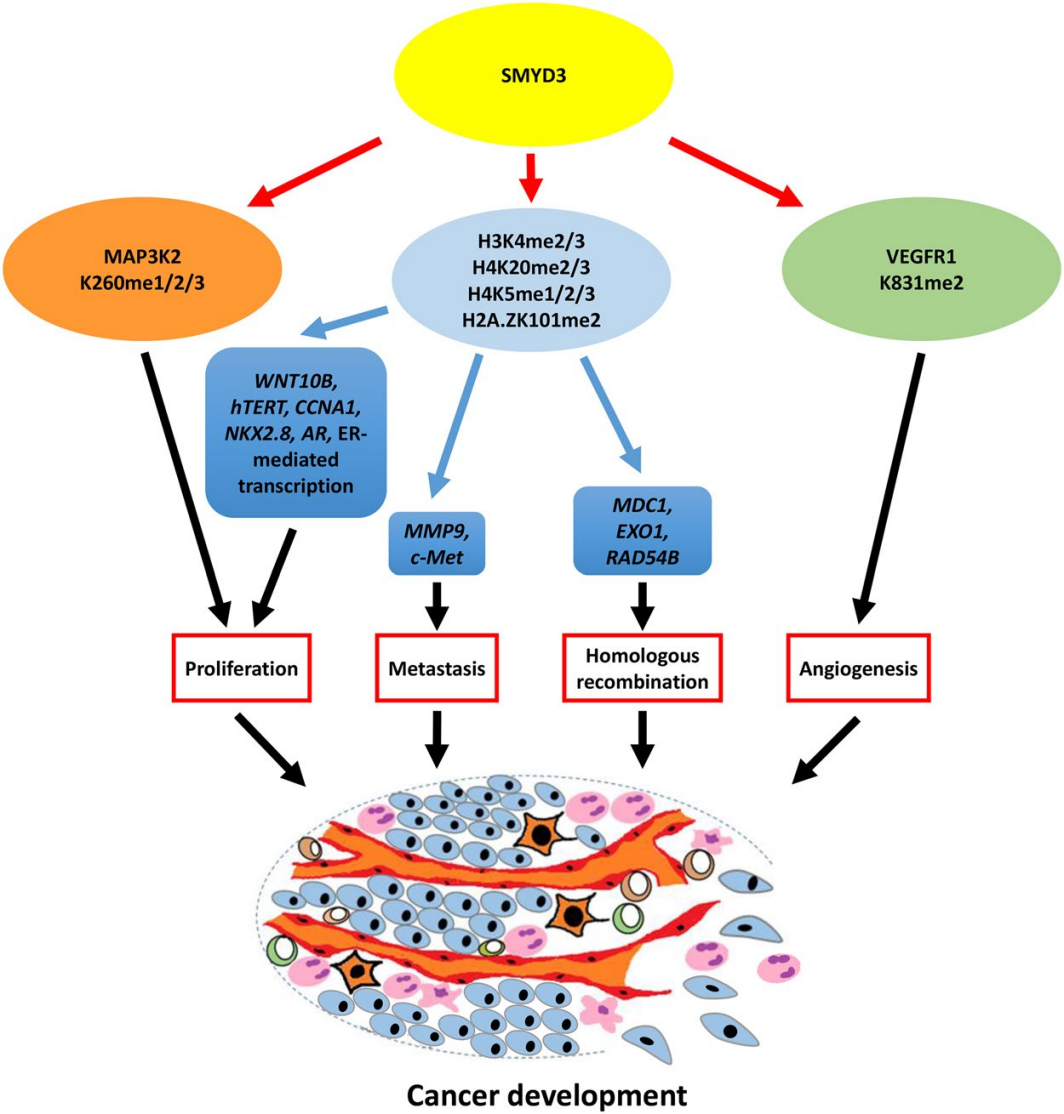
SMYD3 methylates histone and non-histone substrates to regulate different pathways that are important for hallmarks of cancer. SMYD3 directly methylates proteins that are involved in cell proliferation and angiogenesis. Moreover, SMYD3 methylates histones that are widely spread on chromatin to facilitate transcription of target genes. Hence, the high level of SMYD3 stimulates cancer development.

SMYD3 is a transcriptional regulator that functions, in part, through histone methylation to control the expression of target genes. Apart from previously identified targets *WNT10B* ^25^, *hTERT* ^31^, *CCNA1* ^26^, *NKX2*.*8* ^24^, *MMP9* ^46^, and *c*-*MET* ^47^, we show here that SMYD3 facilitates expression of several genes involved in the whole process of HR repair. Deficiency of SMYD3 hampers the expression of these HR associated genes, induces DNA-damage hypersensitivity, causes genomic instability, decreases the levels of MDC1 and BRCA1 foci, and leads to impairment of HR-mediated DSB repair. Furthermore, generation of the active transcription mark H3K4me3 and phosphorylation of RNAPII C-terminus Ser5 at the *MDC1*, *EXO1*, and *RAD54B* promoters are SMYD3-dependent. These findings provide insights into how SMYD3 functions as an oncogene besides its abilities in promoting cell cycle, cell proliferation, and metastasis. The newly identified role of SMYD3 in HR repair proves that SMYD3’s function is crucial in maintaining genome stability (Fig. 6). Consistent with recent reports, highly proliferative cells, such as cancer cells, rely profoundly on HR-mediated DSB repair to restart the stalled replication forks at S phase^48, 49^.

The activity of SMYD3 is closely related to the cell cycle progression^26, 50^. Deficiency of SMYD3 leads to G1-phase cell cycle arrest in breast cancer cells^30, 50^ and S-phase arrest in prostate cancer cells^51^. Therefore, it is likely that slower removal of IR-induced γH2A.X foci (Fig. 1b) might also be contributed partly from the impact of SMYD3 on cell cycle. Notably, SMYD3 deficient cells could proceed the cell cycle to finish mitosis, but generate more micronuclei which represent aberrant chromosomal segregation (Fig. 2d). The combinational effects of genomic instability and the lack of proper DNA repair machinery in SMYD3-depleted cells may therefore lead to apoptosis^50, 51^.

Large-scale screens have revealed a correlation between SMYD3 and DNA repair pathways. Knockdown of SMYD3 caused differentially expressed DNA repair genes *RAD50* and *RAD51* in prostate cancer cells^52^. A proteomic analysis discovered that SMYD families share some common interactors (NPM1, TOP1, GNL3, and RUVBL2) involved in DNA repair and chromatin maintenance^53^, which may imply that SMYD3 not only modulates the DNA repair pathways at the transcription level but also directly interacts with DDR proteins. Moreover, mono-methylation of poly(ADP-ribose) polymerase-1 (PARP1) by SMYD2, another SMYD family methyltransferase, enhances PARP1 activity and cellular response to oxidative DNA damage^54^. Therefore, we propose that SMYD3 may regulate DNA repair at both transcriptional and posttranscriptional levels.

Most histone methylation occurs at specific sites of H3 and H4, which is controlled by a large group of *methyltransferases* and demethylases. For example, SETD2-dependent H3K36me3 is essential for efficient end resection of HR through the recruitment of CtIP, RPA, and RAD51^55, 56^. Interestingly, the levels of SETD2 and H3K36me3 are not induced after DSB damage, suggesting their pre-established role on chromatin^56, 57^. Similarly, the distribution and protein amount of SMYD3 are not adjusted after IR treatment (Supplementary Fig. S1). These finding may imply that the amount of nuclear SMYD3 is sufficient for driving HR gene expression and that these HR proteins are constitutively expressed for taking care of sudden DNA damage. The property that SMYD3 is preferentially recruited to individual promoters may rely on both its binding sequences and its binding partners. Further investigation is required to clarify this specificity.

## Methods

### Cell lines, plasmids, and transfection

The breast cancer cell lines MCF7, MDA-MB-231 and AU565 were maintained in their respective media according to ATCC protocols. HEK293T cells were co-transfected with packaging plasmid (pCMV-Δ8.91), envelope (pMDG), and hairpin pLKO-RNAi vectors (National RNAi Core Facility, Institute of Molecular Biology/Genomic Research Centre, Academia Sinica, Taiwan) for virus packaging. The specific oligo sequences of shRNA are listed in Supplementary Table S1. Virus-containing supernatants were collected at 48 hr post-transfection. Cells were treated with virus plus medium containing polybrene (8 μg/ml) for 16 hr. The infected cells were selected with puromycin (1 μg/ml). Plasmids expressing SMYD3^WT^ and methyltransferase-dead SMYD3^Y239F^ were constructed as previously described^26^.

### Data analysis of gene expression microarray

The microarray analysis of shLuc vs. shSMYD3 viruses-treated MCF7 cells was performed as previously described with the NCBI Gene Expression Omnibus (GEO) accession number GSE58048^26^. The list of down-regulated genes was further categorized by the DAVID v6.8 Gene Ontology program^58, 59^.

### Cell fixation and immunofluorescence assays

Cells were seeded on glass coverslips coated with poly-L-lysine (Sigma-Aldrich, St. Louis, MO, USA) and allowed to attach for 48 hr followed by 1.67 Gy IR treatment (IBL 637, CIS Bio International, Gif-sur-Yvette, France). After washed with phosphate buffered saline (PBS), cells were fixed in 4% paraformaldehyde in PBS for 10 min at room temperature. The fixed cells were then permeabilized with 0.1% Triton X-100 in PBS for 10 min. After 30 min of blocking with 1% BSA in PBS, cells were washed in PBS and incubated with primary antibodies for 3 hr. After three times washed in PBS containing 0.05% Triton-X for 5 min, the cells were incubated with secondary antibodies for 1 hr. Finally, cells were washed three times with PBS containing 0.05% Triton-X and embedded in 1 μg/ml DAPI (Sigma-Aldrich) containing mounting solution on glass slides. The cells were visualised with an Olympus fluorescence microscope. Images were captured using a Spot advanced imaging system. The primary antibodies used were γH2A.X (05-636, Millipore-Upstate, Temecula, CA, USA), BRCA1 (sc-6954, Santa Cruz Biotechnology, CA, USA), 53BP1 (sc-22760, Santa Cruz Biotechnology) and MDC1 (A300-053A, Bethyl Laboratories, Montgomery, TX, USA).

### Colony formation assay

For the colony formation assay, control (shLuc) or knockdown (shSMYD3) cells were seeded (5,000 cells for shLuc cells, and 20,000 and 10,000 cells for shSMYD3#1 and shSMYD3#2 cells, respectively) in 6-cm dishes two days before IR (0, 1.67, 3.34, 5.01 Gy) treatment. Cells were incubated for 15 days, fixed in 4% paraformaldehyde for 5 min, washed once with PBS, stained with 0.1% crystal violet, and then washed with distilled water. The survival rate was calculated by comparing the colonies numbers with the non-irradiated cells in each group.

### Nuclear/cytosol fractionation

Approximate 1 × 10^6^ MCF7 cells were trypsinized and washed with ice-cold PBS twice followed by lysed on ice for 10 min in 250 μl cytoplasmic lysis buffer (0.1% Triton-X, 10 mM HEPES-KOH pH 7.9, 10 mM KCl, 1.5 mM MgCl_2_, 0.34 M sucrose, 10% glycerol) containing protease inhibitor, 1 mM DTT, and 10 mM PMSF. Nuclear sediments were collected by centrifugation at 6,000 rpm for 1 min, and pellets were washed twice with 1 ml cytoplasmic lysis buffer. Nuclei were lysed in RIPA buffer (50 mM Tris-HCl, 150 mM NaCl, 1% NP-40, 0.25% sodium deoxycholate, 1% sodium dodecyl sulfate (SDS), 1 mM DTT, protease inhibitor, 1 mM PMSF, 1 mM EDTA) and lysed completely by sonication. Nuclei and cytosolic extracts were then subjected to Western blot analysis.

### Western blot analysis

Western blot analysis was performed as described^26^. The primary antibodies used were SMYD3 (GTX121945, Genetex, San Antonio, TX, USA), MDC1 (GTX102673, GeneTex), EXO1 (GTX109891, GeneTex), RAD54B (GTX103291, GeneTex), Tubulin (GTX112141, GeneTex), and nuclear matrix protein p84 (NB100-174, Novus, Littleton, CO, USA). The quantification of protein expression was performed using ImageJ software (Image Processing and Analysis in Java). All protein expression levels were normalized against the corresponding control protein levels as indicated. Images were representatives of n ≥ 3 for each experiment.

### Comet assay

DNA strand breaks were evaluated using alkaline single cell gel electrophoresis (comet) assay following the procedure of Olive and Banath^60^. The quantification of tail moment, a representation of the fluorescence intensity in the tail relative to the head, was performed using CometScore (Autocomet.com), with at least 100 individual cells were analysed per condition.

### Micronuclei counts

For micronuclei analysis^34, 35^, cells were seeded and fixed as described in the immunofluorescence assays. The cells were then incubated with SYBR gold (Thermo Fisher Scientific Inc., Waltham, MA, USA) for 1 min, washed twice with PBS, and mounted for microscopy. Objects were defined as micronuclei if they were clearly separated from the nuclei, were round- to oval-shaped with distinct borders, had an area of less than a quarter of the area of a nucleus, and showed staining characteristics similar to those of nuclei.

### RNA analysis and quantitative real-time polymerase chain reaction (qRT-PCR)

Total RNA was isolated using the RNeasy kit (Qiagen, Valencia, CA, USA). RNA was reverse-transcribed into first-strand cDNA using AMV reverse transcriptase (Promega, Madison, WI, USA). cDNA was amplified with KAPA SYBR Fast PCR Mix (KAPA Biosystems, Woburn, MA, USA) and subjected to analysis using a CFX Connect Real-Time System thermal cycler (Bio-Rad, Hercules, CA, USA). *RPL30* mRNA, which encodes the ribosomal protein L30, was used as an internal control. The relative abundance of mRNA was calculated after normalization with *RPL30* mRNA using the CT equation. For verifying candidate genes in microarray data, primers were either designed based on the coding sequence of each genes using Primer3Plus^61^ or directly retrieved from Origene website (http://www.origene.com/). The primers used are listed in Supplementary Table S2.

### HR assay

HR efficiency was measured in MCF7/DR-GFP cells, according to the previous report^62^. The MCF7 DR-GFP cells harbor GFP-based chromosomal reporters. The stable cells possess two differential *GFP* mutant genes oriented as direct repeats and separated by a drug selection marker, the puromycin N-acetyltransferase gene. Transient expression of the I-SceI enzyme produces a DSB in one of the two *GFP* mutant genes. The DSB can be repaired by HR between the two *GFP* mutant genes, resulting in the restoration of a functional *GFP* gene and the expression of GFP proteins. After knockdown of target genes for three days, cells were transfected with pCASce to express the I-SceI protein. GFP-positive cells were measured by flow cytometry (BD FACSCalibur, BD Biosciences, Miami, FL, USA) in 48 hr. Ds-Red was transfected in a parallel group as a control for transfection efficiency.

### Plasmid based end-joining assay

Plasmid end-joining assay was conducted as previously described^63^. The pGL3-promoter plasmid (Promega), which harbors a luciferase reporter gene, was linearized by HindIII and confirmed by agarose gel electrophoresis. The linearized DNA was purified by gel extraction kit (Qiagen), dissolved in sterilized water, and transfected into cells after knockdown of target genes for three days. Luciferase protein was expressed when the cutting sites were repaired by end-joining. The luciferase activity was assayed by Luciferase Assay System (Promega). A Renilla plasmid was co-transfected as a control.

### Plasmid based MMEJ assay

MMEJ efficiency was measured according to the previous report^64^. The pSV40-MMEJ plasmid, which harbors a GFP reporter gene, was a gift from Dr. Nicolas Mermod. Plasmids were linearized by I-SceI, purified by gel extraction kit (Qiagen), and transfected into cells after knockdown of target genes for three days. GFP protein was expressed when the cutting sites were repaired by MMEJ. The pGK-GFP plasmid was transfected in parallel as a transfection efficiency control. Expression of GFP was measured by flow cytometry (FACSCalibur, BD BioSciences) after 48 hr of transfection.

### ChIP assay

ChIP assays were performed as described^26^. Complexes were immunoprecipitated overnight with 2 μg of antibodies specific for SMYD3 (GTX121945, GeneTex), rabbit IgG (GTX35035, GeneTex), H3 (ab1791, Abcam, Cambridge, MA, USA), H3K4me3 (ab10158, Abcam), and RNA polymerase II CTD repeat YSPTSPS (phosphor Ser5) (ab5131, Abcam). Input samples were processed in parallel. Antibody/protein complexes were collected by 40 μl of protein G-coupled Sepharose beads (GE Healthcare Bio-Sciences, Pittsburgh, PA, USA) and washed as follows: once with Tris/EDTA-150 mM NaCl, twice with Tris/EDTA-500 mM NaCl, and once with PBS. Immune complexes were eluted with 1% SDS and TE buffer. After decrosslinking, DNA was purified using a PCR cleanup kit (Qiagen) and analysed by qRT-PCR. The results were expressed as the percentage of the initial inputs. The primer sets used for the ChIP assay are listed in Supplementary Table S2.

### Statistical analysis

Each experiment was repeated at least three times with comparable results. Results are expressed as mean ± SD. All statistical analyses were performed using Excel 2010 (Microsoft; Redmond, WA). The *p*-values for all experiments were obtained using two-tailed Student’s *t* tests to evaluate differences between two groups, with *P* < 0.05 considered statistically significant.

## Electronic supplementary material

Supplementary information
