# Supplementary material for: SMYD3 Promotes Homologous Recombination via Regulation of H3K4-mediated Gene Expression
Source: Sci Rep. 2017 Jun 19;7:3842. doi: 10.1038/s41598-017-03385-6 (PMC5476597; doi:10.1038/s41598-017-03385-6)
Supplement: Supplementary file 1 — Supplementary information [file 41598_2017_3385_MOESM1_ESM.pdf]

SMYD3 Promotes Homologous Recombination via Regulation of H3K4-mediated Gene Expression

Yun-Ju Chen[1], Cheng-Hui Tsai[1], Pin-Yu Wang[1], and Shu-Chun Teng[1,2,3]

[1]Department of Microbiology, College of Medicine, National Taiwan University, Taipei, 10051, Taiwan
[2]Ph.D. Program in Translational Medicine, National Taiwan University and Academia Sinica, Taipei, 10051, Taiwan
[3]Address correspondence to Department of Microbiology, College of Medicine, National Taiwan University, No. 1, Sec. 1, Jen-Ai Road, Taipei 10051, Taiwan
Phone: (886) 2-23123456 ext. 88289
Fax: (886) 2-23915293
E-mail: shuchunteng@ntu.edu.tw

Keywords: SMYD3, methyltransferase, homologous recombination, H3K4, cancer

Supplementary Information

Supplementary Figures, Supplementary Figure legends and Supplementary Tables.

Supplementary Figure S1. SMYD3 location and expression are not modulated by DNA damage insults.
Supplementary Figure S2. The knockdown efficiency of each knockdown clones used and Western blotting for protein expression levels in Fig 3.
Supplementary Figure S3. The formation of DNA repair foci.
Supplementary Figure S4. SMYD3 activates the expression of *EXO1* and *RAD548* through methylating histone H3K4.

Supplementary Table S1. Oligo sequences for shRNA-mediated gene knockdown.
Supplementary Table S2. Primers used in this study.

Supplementary Figure S1.

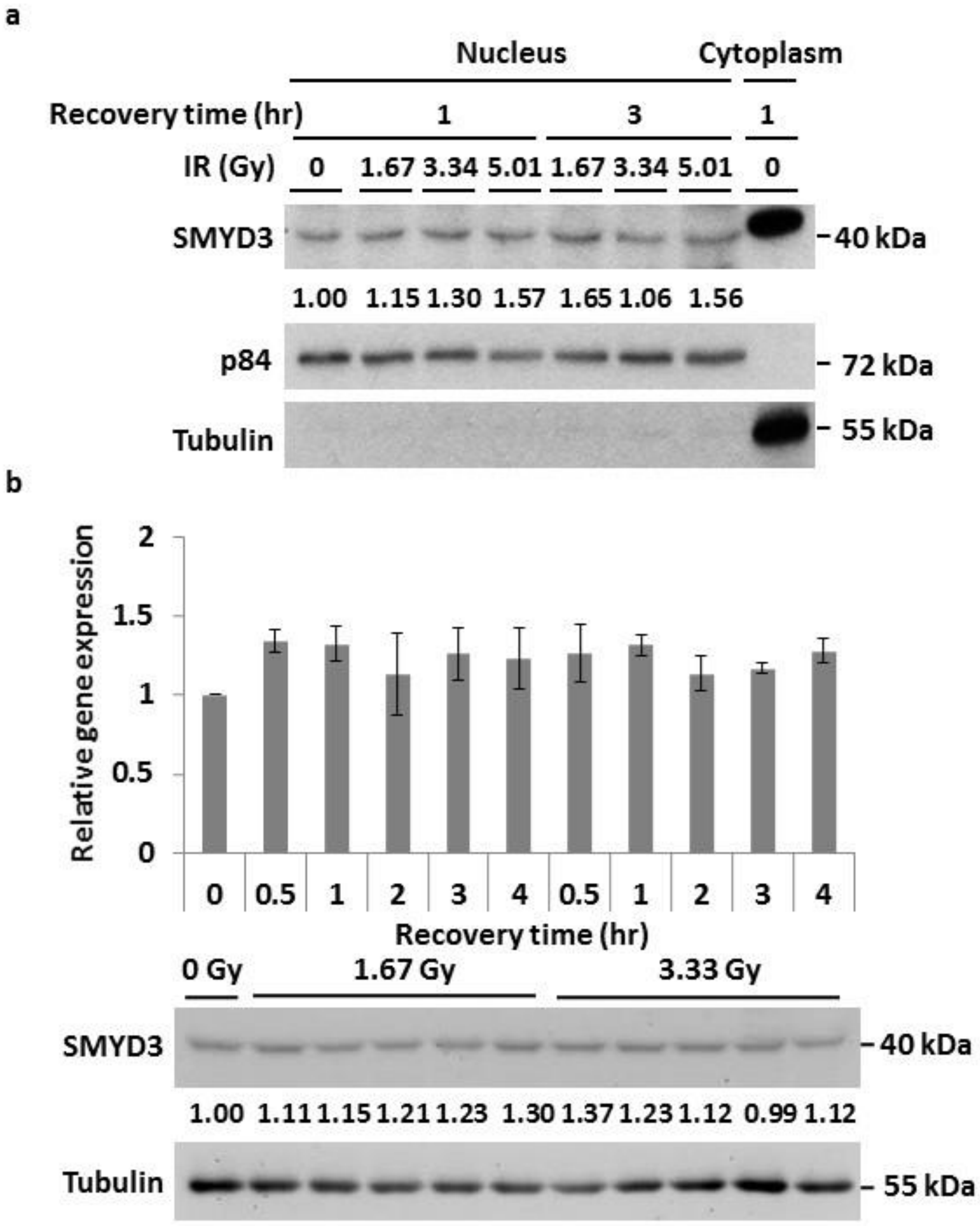

Supplementary Figure S1. SMYD3 location and expression are not modulated by DNA damage insults.
(a) The amounts of SMYD3 in the nucleus was analysed by nuclear/cytosol fractionation at 1 hr and 3 hr post-IR with different dosages. Western blot analyses were performed using indicated antibodies. p84 was used as a nuclear-specific marker for the loading control. Tubulin was used to check the purity of nuclear fractions. **(b)** SMYD3 expression levels at 1 hr and 3 hr post-IR with different dosages. Upper panel, the mRNA expression levels of SMYD3 were examined by qRT-PCR. Lower panel, the protein expression levels of SMYD3 were detected by Western blot analyses. All values in the histograms were means ± SD of triplicates and data were representative of $n \geq 3$ for each experiment.

Supplementary Figure S2.

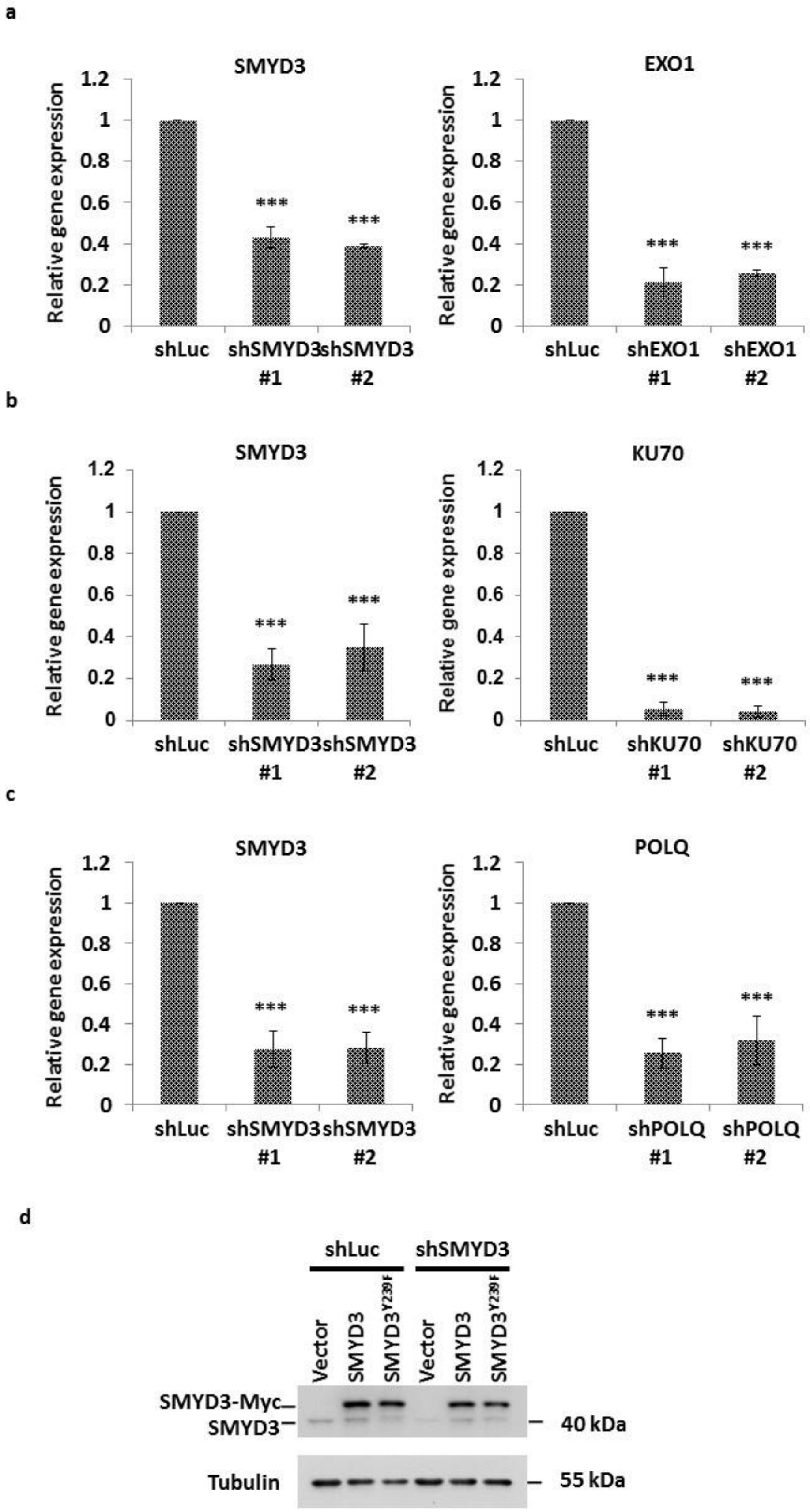

Supplementary Figure S2. The knockdown efficiency of each knockdown clones used and Western blotting for protein expression levels in Fig 3.

The knockdown efficiency was validated by qRT-PCR six days after selected with puromycin. **(a)** Knockdown efficiency of *SMYD3* and *EXO1* in MCF7/DR-GFP cells. **(b)** Knockdown efficiency of *SMYD3* and *Ku70* in MCF7 cells. **(c)** Knockdown efficiency of *SMYD3* and *POLQ* in MCF7 cells. **(d)** Western blotting of cells subjected to Luc or SMYD3 knockdown and complemented with vector control or Myc-tagged SMYD3 or mutant $SMYD3^{Y239F}$. ***, $P < 0.001$ vs. shLuc control. All values in the histograms were means ± SD of triplicates and data were representative of $n \geq 3$ for each experiment.

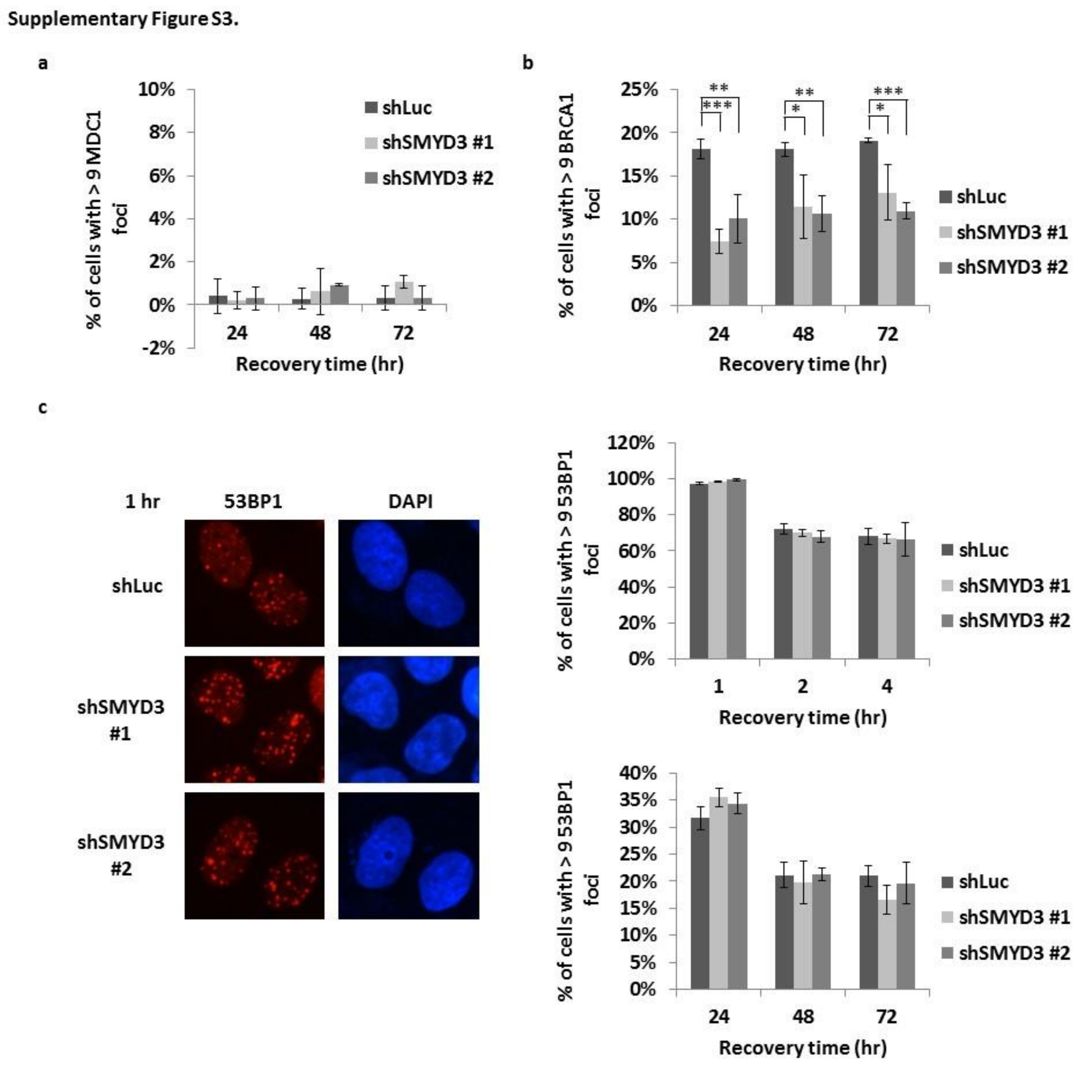

Supplementary Figure S3. The formation of DNA repair foci.
MDC1 foci **(a),** BRCA1 foci **(b)**, and 53BP1 foci formation **(c)** at indicated times after 1.67 Gy IR treatment in shLuc or shSMYD3 MCF7 cells. *, $P < 0.05$. **, $P < 0.01$. ***, $P < 0.001$ vs. shLuc control. All values in the histograms were means ± SD of triplicates and data were representative of n ≥ 3 for each experiment.

Supplementary Figure S4

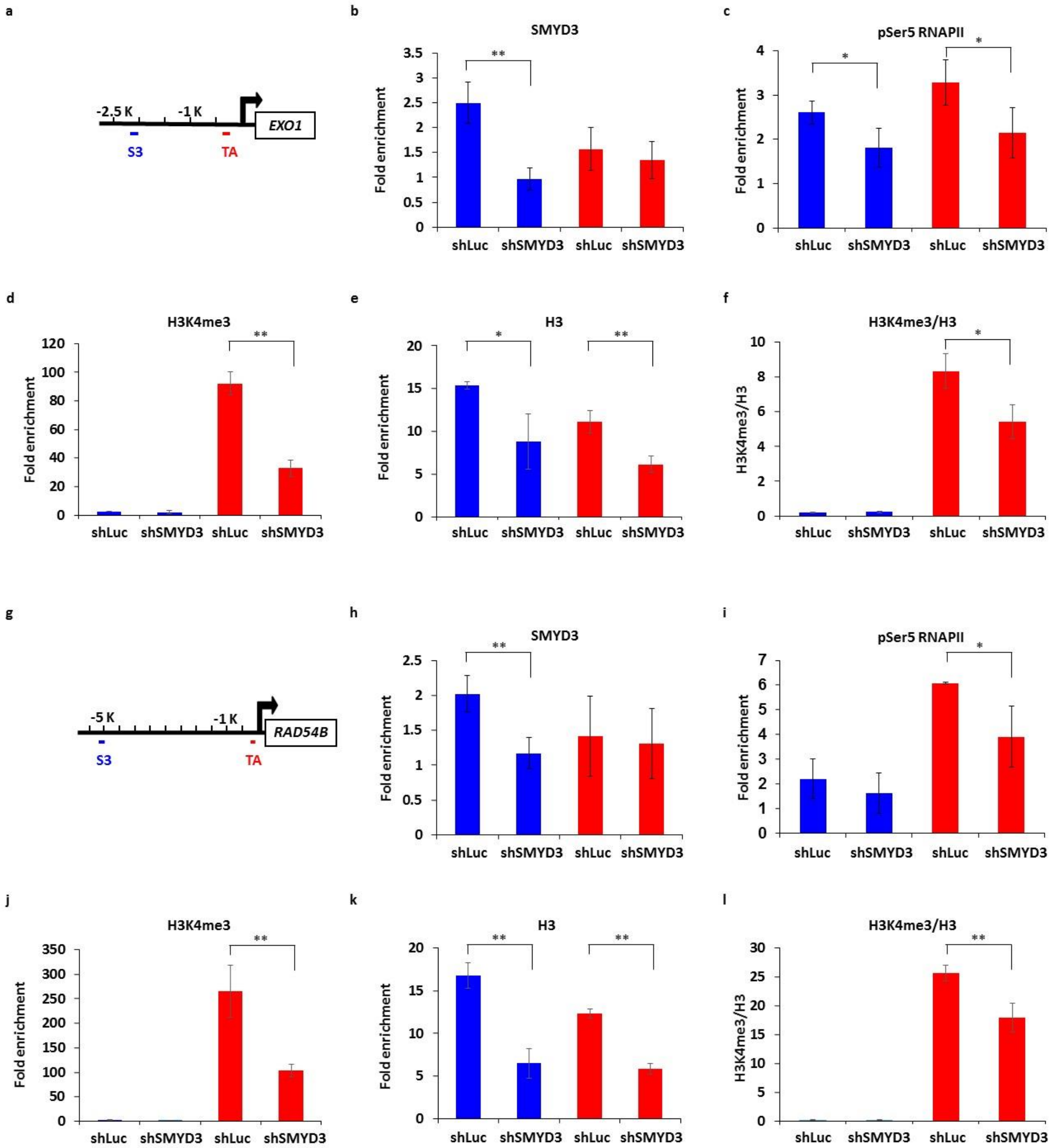

Supplementary Figure S4. SMYD3 activates the expression of *EXO1* and *RAD548* through methylating histone H3K4.
(a and g) ChIP assays were performed in MCF7 cells using specific antibodies. The examined positions at *EXO1* and *RAD54B* loci were indicated, in which region S3 and region TA are predicted SMYD3 and TATA box binding sites, respectively. **(b-e and h-k)** ChIP assays were performed with SMYD3-repressed MCF7 cells using specific antibodies indicated at the top of the histogram. Fold enrichment of each antibody compared with IgG was shown. **(f and l)** Ratios of H3K4me3/H3 ChIP signals were shown. In **b-f** and **h-l**, immunoprecipitated chromatin was quantified by qRT-PCR. *, $P < 0.05$. **, $P < 0.01$. All values in the histograms were means ± SD of triplicates and data were representative of n ≥ 3 for each experiment.

Supplementary Table S1. Oligo sequences for shRNA-mediated gene knockdown.

Clone ID	Gene Symbol	Target Sequence	Region
TRCN0000123290	*SMYD3*	GCTTCCCGATATCAACATCTA	CDS
TRCN0000123291	*SMYD3*	CAACTCTTTCACCATCTGTAA	CDS
TRCN0000331178	*EXO1*	TGCAGACTGCTGCAAAGCTTT	3'UTR
TRCN0000010332	*EXO1*	AATGCAGACTGCTGCAAAGCT	CDS
TRCN0000332901	*XRCC6 (KU70)*	GATGAGTCATAAGAGGATCAT	CDS
TRCN0000332902	*XRCC6 (KU70)*	CCCAAGGTTGAAGCAATGAAT	CDS
TRCN0000290546	*POLQ*	CCTTCAATCTTGCTTGCGAAA	CDS
TRCN0000290546	*POLQ*	GCTGACCAAGATTTGCTATAT	CDS

Supplementary Table S2. Primers used in this study.

Genes	Forward sequence	Reverse sequence	Assay
SMYD3	TTACTGCGAGCAGTCCGAGACA	TTGTCCTGGGTTTGGCAACGGA	SYBR green qPCR
TRIP13	CAGCAGCACTGCAAAGAAAG	AAATCGATGGGCTGTGAGTC	SYBR green qPCR
MDC1	GCAAGATGCCACCTGCTGAGAA	GCTTCAGGTACTGTAGGAGGCA	SYBR green qPCR
BARD1	TGTGGTTTAGCCCTCGAAGT	GCCCTCTCAGAAACATCTGC	SYBR green qPCR
UHRF1	TGTGGACCATGGGAATTTTT	GGGAGCAAAGCAGTTGAGAG	SYBR green qPCR
FANCD2	TTCCAGGATGCCTTCGTAGTGG	GCAGGAGGTTTATGGCAATCCC	SYBR green qPCR
BRCA2	GGCTTCAAAAAGCACTCCAGATG	GGATTCTGTATCTCT TGACG TTCC	SYBR green qPCR
EXO1	TCGGATCTCCTAGCTTTTGGCTG	AGCTGTCTGC ACATT CCTAG CC	SYBR green qPCR
RAD18	GGATTGTCCTGTTTGCGGGGTT	GTTTTGGGCA GCGGC TTCCT TT	SYBR green qPCR
RAD51	TCTCTGGCAGTGATGTCCTGGA	TAAAGGGCGG TGGCA CTGTC TA	SYBR green qPCR
CHEK1	GTGTCAGAGTCTCCCAGTGGAT	GTTCTGGCTG AGAAC TGGAG TAC	SYBR green qPCR
LIG1	TCACAGAGGCTGAAGTGGC	TCAGGCTCTG AAACG CTTTC CG	SYBR green qPCR
RAD51AP1	CTTCTGGAAGGCAGTGATGGTG	AGAGAAGTCTTCGTCATTATCCTC	SYBR green qPCR
RAD54B	TCATGATCTG CTTGA CTGTG AG	TTTTTCCAACGAATCACCTGT	SYBR green qPCR
KU70	TGCCACAGGA AGAAG AGTTG	CTCTG GAGTT GCCAT GATTT	SYBR green qPCR
POLQ	CTTGTGGCAT CTCCT TGGAG CA	AATCC CTTGG CTGGT CTCCA TC	SYBR green qPCR
MDC1	CCTCTCAAAGTGGTGGGATT	AATTGCTTGAACCCAGAAGG	Region S3: -530~-377 bp
MDC1	AGGAGAATCGCTTGAACCTG	CTTAAAGGCTGTCCCCACCT	Region TA: -234~-49 bp
EXO1	TCACCTGAGGTTGGGAGTTC	ACTGCAACCTCTGCCTCCT	Region S3: -2278~-2114 bp
EXO1	AAGGCCCATTTTCAAGGTCT	ATTCAGTTCACGCTGGGTTC	Region TA: -386~-237 bp
RAD54B	AGACCTCCCCAGATGATTCC	CCCGAATAGCTGGGACTACA	Region S3: -5262~-5049 bp
RAD54B	TTCGTTTCTATATTCCCAGAACCT	ATGATTCGGTGTGTGCGATA	Region TA: -398~-247 bp
